# Response to comment on: [Potential bias in the analysis of prenatal treatment for congenital toxoplasmosis]

**DOI:** 10.1371/journal.pntd.0013138

**Published:** 2025-07-07

**Authors:** Borja Guarch-Ibáñez, Clara Carreras-Abad, Marie Antoinette Frick, Daniel Blázquez-Gamero, Fernando Baquero-Artigao, Isabel Fuentes, Pere Soler-Palacin

**Affiliations:** 1 Pediatric Infectious Diseases Unit, Pediatrics Department, Hospital Universitari de Girona Dr. Josep Trueta, Girona, Catalonia, Spain; 2 Universitat de Girona (UDG), Girona, Catalonia, Spain; 3 Congenital Infections Working Group, Spanish Society of Pediatric Infectious Diseases (SEIP), Spain; 4 Pediatric Infectious Diseases Unit, Pediatrics Department, Hospital Universitari Germans Trias i Pujol, Badalona, Catalonia, Spain; 5 Pediatric Infectious Diseases and Immunodeficiencies Unit, Hospital Infantil Vall d’Hebron, Barcelona, Catalonia, Spain; 6 Vall d’Hebron Research Institute, Barcelona, Catalonia, Spain; 7 Pediatric Infectious Diseases Unit, Hospital Universitario 12 de Octubre, Madrid, Spain; 8 Pediatric Infectious Diseases Unit, Hospital Universitario La Paz, Madrid, Spain; 9 Universidad Autónoma de Madrid, Madrid, Spain; 10 CIBERINFEC, Instituto de Salud Carlos III, Madrid, Spain; 11 Toxoplasmosis and Intestinal Protozoa Unit, Centro Nacional de Microbiología, Instituto de Salud Carlos III, Madrid, Spain; Centro de Pesquisa Gonçalo Moniz-FIOCRUZ/BA, BRAZIL

We sincerely appreciate the authors’ thoughtful commentary on our recently published article. Their valuable contribution enriches the quality of the ongoing debate on congenital toxoplasmosis (CT). We also welcome the opportunity to respond to their formal comment on our study. In this regard, we would like to address certain aspects raised, particularly concerning potential biases related to prenatal screening, the implications of excluding severe cases, and the interpretation of the SYROCOT study.

It is true that our study has limitations, as almost all previously published studies in this topic, and these were all already noted in the published article. The REIV-TOXO cohort is based on a voluntary registry, thus limiting its representativeness with some CT cases potentially not reported. Also, data on abortions, premature births, and co-infections were not included, and the low rate of brain MRIs (19/54) could underestimate CNS involvement. Furthermore, the study design did not allow to investigate any possible association adjusted for the ‘window of opportunity,’ nor did it assess the emergence of new infection-related complications during follow-up by controlling for the effect of postnatal treatment. Despite these limitations, we strongly believe that its results clearly support the effectiveness of prenatal treatment and pushes the need for an active debate on this topic.

Our study evaluates the effect of prenatal treatment on CT by comparing treated versus untreated children, irrespective of whether prenatal screening was performed. The concern raised about potential bias due to prenatal screening suggests that differences in screening practices might introduce bias by overestimating the treatment effect. However, our analysis does not compare screened versus non-screened cases; instead, it directly evaluates clinical outcomes at birth and during two years of follow-up in children who received prenatal treatment versus those who did not. This approach ensures that our conclusions accurately reflect the efficacy of prenatal treatment, independent of screening variability across hospitals.

Moreover, postnatally diagnosed cases should not be excluded from the analysis, as doing so would underestimate the potential effect of prenatal treatment on symptomatic cases identified after birth.

In reference to the statement regarding the SYROCOT study, we are aware that this retrospective, multicenter, European study did not find that prenatal treatment reduced the risk of neonatal symptoms. However, this study had significant limitations that affected its results: severe fetal cases were excluded aiming to avoid “referral bias”; the final analysis did not include a control group (untreated pregnant women) because of ethical concerns (implying the idea of the benefit of treatment); and neonatal follow-up was limited to only one year. In fact, when a subgroup of SYROCOT investigators analyzed the data that included cases with severe neurologic sequelae or death, prenatal treatment (either spiramycin and/or pyrimethamine-sulfadiazine) was associated with a reduced risk of severe neurologic sequelae or death.[1] The conclusions of the SYROCOT study are in line with a series of articles published between 1999 and 2007 that, unfortunately, were biased, misleading, and failed to accurately reflect the biological impact of prenatal treatment.[2–3] In contrast, a recently published study by Gomes Ferrari Strang AG et al.[4], consistent with our findings and with other studies cited in our paper in support of prenatal treatment, showed that children born to untreated mothers had a 6.5-fold higher risk of infection compared to those whose mothers received treatment.

In conclusion, recent evidence strengthens the argument that prenatal treatment may not only reduce maternal-fetal transmission but also mitigate the severity of sequelae in infected children. However, further high-quality research is needed to determine the optimal approach to managing CT.
